# Transcriptional Analysis Implicates Endoplasmic Reticulum Stress in Bovine Spongiform Encephalopathy

**DOI:** 10.1371/journal.pone.0014207

**Published:** 2010-12-03

**Authors:** Yue Tang, Wei Xiang, Linda Terry, Hans A. Kretzschmar, Otto Windl

**Affiliations:** 1 Department of Molecular Pathogenesis and Genetics, Veterinary Laboratories Agency, Surrey, United Kingdom; 2 Institute of Biochemistry, Emil-Fischer-Center, University Erlangen-Nuernberg, Erlangen, Germany; 3 Institute of Neuropathology, Ludwig-Maximilians-University Munich, Munich, Germany; University of Edinburgh, United Kingdom

## Abstract

Bovine spongiform encephalopathy (BSE) is a fatal, transmissible, neurodegenerative disease of cattle. To date, the disease process is still poorly understood. In this study, brain tissue samples from animals naturally infected with BSE were analysed to identify differentially regulated genes using Affymetrix GeneChip Bovine Genome Arrays. A total of 230 genes were shown to be differentially regulated and many of these genes encode proteins involved in immune response, apoptosis, cell adhesion, stress response and transcription. Seventeen genes are associated with the endoplasmic reticulum (ER) and 10 of these 17 genes are involved in stress related responses including ER chaperones, Grp94 and Grp170. Western blotting analysis showed that another ER chaperone, Grp78, was up-regulated in BSE. Up-regulation of these three chaperones strongly suggests the presence of ER stress and the activation of the unfolded protein response (UPR) in BSE. The occurrence of ER stress was also supported by changes in gene expression for cytosolic proteins, such as the chaperone pair of Hsp70 and DnaJ. Many genes associated with the ubiquitin-proteasome pathway and the autophagy-lysosome system were differentially regulated, indicating that both pathways might be activated in response to ER stress. A model is presented to explain the mechanisms of prion neurotoxicity using these ER stress related responses. Clustering analysis showed that the differently regulated genes found from the naturally infected BSE cases could be used to predict the infectious status of the samples experimentally infected with BSE from the previous study and vice versa. Proof-of-principle gene expression biomarkers were found to represent BSE using 10 genes with 94% sensitivity and 87% specificity.

## Introduction

Transmissible spongiform encephalopathies (TSEs), also termed prion diseases, are fatal, neurodegenerative diseases including Creutzfeldt-Jakob disease (CJD) in humans, scrapie in goats and sheep and bovine spongiform encephalopathy (BSE) in cattle [1], [2], [3], [4]. The infectious agent of these diseases is thought to be an abnormally folded isoform (PrP^Sc^) of the cellular prion protein (PrP^C^) and it is further thought that the accumulation of the misfolded prion protein leads to disease [1]. PrP^Sc^ is characterized by a high β-sheet content and resistance to protease treatment. In addition to the accumulation of PrP^Sc^, the pathological features of prion diseases in the brain of affected subjects include neuronal cell loss and vacuolation. Prion diseases have long incubation periods prior to the onset of clinical signs.

BSE was first discovered in 1986 [5] and became a major epidemic in the UK, peaking in 1992; to date more than 185,000 cases have been recorded. It is thought to be caused by contaminated meat and bone meal, a dietary supplement for cattle [6]. The BSE strain has also most probably crossed the species barrier to humans and has produced variant CJD [7]. The mean incubation period of BSE in cattle is estimated at about 5 years [3]. The clinical signs are: difficulties in locomotion and behavioural changes. The neuropathology of BSE is characterized by the lesions mainly found in the brain stem where vacuolar changes are found in neurons and the neuropil [5]. However, apoptosis plays a very limited role in neuronal loss in BSE [8].

In recent years atypical bovine spongiform encephalopathy has been identified [9], [10]. In these cases the distribution of PrP^Sc^ in the animal differs from that of BSE; there is less PrP^Sc^ accumulation in the brain stem and the biochemical signature of PrP^Sc^ is different.

The pathogenesis of BSE is still poorly understood. In a previous gene expression study using brain tissue samples from cattle experimentally infected with BSE, we have demonstrated that the largest number of differentially regulated genes is detected at 21 months post inoculation, suggesting that there are many pathogenic processes in the animal brain even prior to the detection of infectivity in the CNS of these orally dosed cattle [11]. Moreover, a set of differentially regulated genes could be used to predict the infectious status of preclinical samples.

To further understand the pathogenesis of BSE and to explore the possibility of using gene expression profiles as biomarkers, we analysed brainstem RNA samples from confirmed naturally infected cases of BSE (field cases) in cattle and from healthy controls.

## Results

### Identification of differentially regulated genes in the BSE field case samples

The expression of genes in the brain of naturally infected BSE samples was compared with negative controls. In order to identify differentially regulated genes in BSE, the following stringent conditions were set with two filters: 2 fold change and one-way ANOVA with the p value being 0.05. 409 probe sets (a technical term that describes a transcript on the microarray) were identified as differentially regulated between the BSE infected (n = 14) and negative (n = 12) samples. After removal of duplications, 230 genes were identified and these genes are listed in Table 1 and the unannotated probe sets are listed in Table S1.

**Table 1:** Relative levels of differentially expressed genes of BSE.

	Gene ID	Gene Nams	Fold	p-value
			change	
**Cell adhesion**	Bt.23129.3.S1	similar to Laminin gamma-1 chain precursor (Laminin B2 chain)	2.70	0.0209
	Bt.2573.1.S1	CD9 antigen (p24)	3.61	0.0164
	Bt.4817.2.S1	claudin 11	5.38	0.019
	Bt.8382.2.S1	ras homolog gene family, member B	2.14	0.0119
	Bt.11224.1.S1	similar to 85 kDa lysosomal sialoglycoprotein	2.83	0.0266
	Bt.15742.1.S2	CD47 molecule	2.04	0.0316
	Bt.18378.1.S1	similar to KIAA1014 protein	2.29	0.0401
	Bt.4653.1.S2	platelet/endothelial cell adhesion molecule	3.57	0.0399
**Apoptosis**	Bt.5250.1.S1	milk fat globule-EGF factor 8 protein	2.58	0.035
	Bt.222.1.S1	crystallin, alpha B	2.07	0.0469
	Bt.13130.1.S1	tumor necrosis factor receptor superfamily, member 5	−2.33	0.0469
	Bt.16079.1.S1	**reticulon 3** *	2.10	0.041
	Bt.21430.1.S1	similar to Synovial apoptosis inhibitor 1, synoviolin	5.18	0.0237
	Bt.16916.1.S1	TGF-beta inducible early growth response protein 2	2.24	0.028
	Bt.23228.1.S1	Similar to Fas apoptotic inhibitory molecule 2	2.74	0.041
	Bt.2408.1.S1	chemokine (C-C motif) ligand 2	−3.07	0.0428
	Bt.8220.1.A1	similar to transforming acidic coiled coil 1	2.21	0.0242
**Immune**	Bt.9504.1.A1	putative MIP1-beta protein	−4.55	0.0239
**responses**	Bt.24900.1.S1	similar to T-cell immunomodulatory protein	2.2	0.0141
	Bt.29761.1.S1	T-cell receptor beta chain variable segment	−4.69	0.0172
	Bt.26847.1.S1	linker for activation of T cells	−2.75	0.0423
	Bt.4060.1.S1	T-cell differentiation protein Mal	2.87	0.0109
	Bt.4175.2.S1	similar to minor histocompatibility antigen 13	3.69	0.0467
	Bt.3791.1.S1	basigin	2.11	0.0401
**cell cycle &**	Bt.22534.1.S1	similar to peripheral myelin protein 22	2.69	0.0171
**growth**	Bt.11059.1.S1	Putative tumor suppressor LUCA15) (G15 protein	2.85	0.0084
	Bt.2214.1.S1	similar to prostacyclin-stimulating factor; PGI2-stimulating factor; PSF	2.09	0.0164
	Bt.2220.2.A1	selenoprotein P, plasma, 1	2.45	0.0171
	Bt.4750.1.S1	transketolase	2.04	0.0219
	Bt.29157.1.A1	growth arrest-specific 2 like 1	−2.58	0.0438
	Bt.29718.2.A1	growth hormone receptor	−4.46	0.0129
	Bt.51.1.S1	cyclin-dependent kinase 5	3.60	0.049
**Extracellular**	Bt.23250.6.A1	alpha-2-HS-glycoprotein	5.68	0.0249
	Bt.28584.1.S1	canopy 3 homolog	3.97	0.0288
	Bt.5313.1.S1	matrix metallopeptidase 2	2.90	0.0427
**cell proliferation**	Bt.4529.1.S1	farnesyltransferase, CAAX box, beta	2.65	0.0119
**& differentiation**	Bt.5224.1.S1	dihydropyrimidinase-like 2	2.52	0.0172
	Bt.435.1.S1	TIMP metallopeptidase inhibitor 2	2.91	0.0069
	Bt.1537.1.S1	N-myc downstream regulated gene 1	2.18	0.0261
**Transport**	Bt.10135.1.A1	similar to solute carrier family 35, member A5	2.76	0.0138
	Bt.13535.1.A1	similar to hippocampus abundant transcript-like 1	2.42	0.0289
	Bt.15466.1.A1	unc-50 homolog	2.78	0.0475
	Bt.26510.1.S1	Proteolipid protein	2.08	0.0264
	Bt.23637.1.S1	adaptor-related protein complex 3, mu 1 subunit	2.08	0.0375
	Bt.21740.1.S1	**transmembrane emp24-like trafficking protein 10**	2.14	0.0499
	Bt.13583.1.A1	similar to ATP-binding cassette transporter G1	2.14	0.0484
	Bt.903.1.S1	similar to choline transporter-like protein 1, splice	2.94	0.0052
	Bt.21168.1.A1	synaptophysin-like 1	2.02	0.0201
	Bt.16001.1.S1	similar to sterol 27-hydroxylase	2.16	0.0272
	Bt.3418.1.S1	mitochondrial carrier homolog 1	2.15	0.0447
	Bt.15804.1.S1	similar to chloride channel protein 3	2.22	0.0175
	Bt.20007.1.S1	ATP-binding cassette, sub-family C (CFTR/MRP), member 5	2.12	0.0471
	Bt.21424.1.A1	similar to receptor Pit2	2.14	0.041
	Bt.22735.1.S1	similar to synaptotagmin-like 2	2.27	0.022
	Bt.2331.1.A1	similar to receptor activity-modifying protein 1	2.15	0.0145
	Bt.23500.1.S1	secretory carrier membrane protein 4	2.23	0.024
	Bt.23518.2.S1	similar to tetracycline transporter-like protein	2.07	0.0122
	Bt.23606.1.S1	**inositol 1,4,5-triphosphate receptor, type 1**	3.53	0.0455
	Bt.269.1.S1	ATPase, Ca++ transporting, type 2C, member 1	2.03	0.0312
	Bt.26994.1.A1	potassium voltage-gated channel, Shal-related subfamily, member 2	−3.54	0.0298
	Bt.27129.1.S1	similar to solute carrier family 39 (zinc transporter), member 9	2.04	0.0335
	Bt.3414.3.A1	HIV-1 Rev binding protein	2.12	0.0312
	Bt.4335.1.S1	similar to protoporphyrinogen oxidase	3.57	0.0204
	Bt.4430.1.S2	similar to vacuolar H+-ATPase subunit	2.09	0.0349
	Bt.4977.1.S2	insulin-like growth factor 2 receptor	2.07	0.0399
	Bt.5000.1.S1	**coatomer protein complex, subunit gamma 2**	2.00	0.0416
	Bt.5293.1.S1	ATPase, H+ transporting, lysosomal 16 kDa, V0 subunit c	2.5	0.0428
	Bt.5293.2.A1	**proteolipid protein 1**	2.4	0.0141
	Bt.5336.1.A1	transferrin	3.22	0.0084
	Bt.6096.1.S1	similar to Conserved oligomeric Golgi complex component 2	5.41	0.0171
	Bt.7134.1.S2	glycolipid transfer protein	2.38	0.0143
	Bt.8822.1.A1	similar to inward rectifier potassium channel Kir1.2	2.76	0.0203
	Bt.9853.1.S1	similar to Solute carrier family 25 member 14	4.35	0.0324
	Bt.26889.1.S1	solute carrier family 33 (acetyl-CoA transporter), member 1	2.11	0.039
	Bt.4646.1.S1	solute carrier family 2 (facilitated glucose transporter), member 1	2.35	0.0171
	Bt.3208.1.S1	DDHD domain containing 2	2.0	0.0306
	Bt.10202.1.S1	**reticulon 4**	2.53	0.024
	Bt.5073.1.S1	transmembrane emp24 protein transport domain containing 4	3.91	0.0499
**Proteolysis**	Bt.20121.1.S1	cathepsin D	2.81	0.0164
	Bt.20030.1.S1	calpain 7	2.25	0.0315
	Bt.12302.1.S1	plasminogen activator, tissue	2.59	0.0203
	Bt.23840.1.S1	similar to subtilisin-like proprotein convertase 4	4.35	0.0175
	Bt.289.1.S1	pregnancy-associated glycoprotein 16	−4.53	0.0475
	Bt.3888.1.S1	protein phosphatase methylesterase 1	3.77	0.0427
	Bt.393.1.S1	cathepsin B	2.18	0.0401
	Bt.5462.1.S2	similar to dynein, cytoplasmic, heavy polypeptide 1	2.1	0.0349
	Bt.1613.1.S1	protease, serine, 11	2.6	0.0373
	Bt.7240.1.S1	leucine aminopeptidase 3	2.65	0.0344
	Bt.27314.1.A1	Similar to Cgi67 serine protease	2.17	0.0427
**Signal transduction**	Bt.5546.1.S1	guanine nucleotide binding protein (G protein), alpha inhibiting activity polypeptide 1	2.02	0.0267
	Bt.9163.1.A1	purinergic receptor P2Y, G-protein coupled, 10	−2.28	0.0249
	Bt.21275.1.S1	splA/ryanodine receptor domain and SOCS box containing 3	4.90	0.0351
	Bt.27421.1.S1	rho/rac guanine nucleotide exchange factor (GEF) 2	2.78	0.0303
	Bt.2235.1.S2	GDP dissociation inhibitor 1	2.07	0.0416
	Bt.24236.1.S1	deleted in liver cancer 1	2.43	0.0226
	Bt.12694.1.S1	similar to Tumor necrosis factor receptor superfamily member 21 precursor (TNFR-related death receptor-6) (Death receptor 6)	3.53	0.0242
	Bt.21758.1.A1	Down syndrome critical region gene 1-like 1	2.77	0.0194
	Bt.20511.1.S1	similar to Ral guanine nucleotide dissociation stimulator A	2.15	0.0175
	Bt.26841.1.A1	GTPase activating Rap/RanGAP domain-like 3	2.02	0.0069
	Bt.2846.1.A1	similar to ras homolog gene family, member U	2.7	0.0175
**Ubiquitin cycle**	Bt.2211.1.S1	ubiquitin-activating enzyme E1 (A1S9T and BN75 temperature sensitivity complementing)	2.19	0.0421
	Bt.23266.1.S1	WW domain containing E3 ubiquitin protein ligase 2	2.26	0.0312
	Bt.5408.1.A1	ubiquitin carboxyl-terminal esterase L1 (ubiquitin thiolesterase)	3.19	0.0226
	Bt.20361.1.S1	F-box and leucine-rich repeat protein 20	2.28	0.0356
	Bt.13185.1	ubiquitin-like modifier activating enzyme 2	2.0	0.0209
	Bt.3753.1.S1	similar to KIAA0614 protein	2.43	0.0477
	Bt.7651.1.S1	ankyrin repeat and SOCS box-containing 11	2.61	0.0476
**Lipid metabolic process**	Bt.4040.1.S1	platelet-activating factor acetylhydrolase, isoform Ib, gamma subunit 29 kDa	2.18	0.0119
	Bt.1229.1.S1	apolipoprotein A-I	3.72	0.0138
	Bt.5467.1.S1	prosaposin	4.07	0.0242
	Bt.6334.1.A1	degenerative spermatocyte homolog 1, lipid desaturase	2.63	0.0203
	Bt.12718.1.A1	Similar to Apolipoprotein D precursor (Apo-D)	3.75	0.0119
	Bt.19709.1.S1	**LAG1 homolog, ceramide synthase 2**	2.71	0.016
	Bt.2342.1.S1	similar to phosphatidate cytidylyltransferase 2	2.02	0.023
	Bt.18340.1.A1	similar to choline/ethanolaminephosphotransferase 1	3.06	0.007
**protein folding**	Bt.23161.2.A1	heat shock 70 kDa protein 1A	4.61	0.0275
	Bt.6149.1.S1	**glucose-regulated protein 170**	2.3	0.0203
	Bt.8686.1.S1	**glucose-regulated protein 94**	2.33	0.0311
	Bt.514.1.S1	DnaJ (Hsp40) homolog, subfamily C, member 6	2.34	0.0242
**Kinase**	Bt.1020.1.S1	similar to CDC-like kinase 1	2.35	0.0483
	Bt.9070.2.S1	centaurin, alpha 1	2.54	0.0209
	Bt.16200.1.A1	WNK lysine deficient protein kinase 2	−2.8	0.0319
	Bt.13980.1.A1	Creatine kinase, mitochondrial 2	2.08	0.0237
	Bt.19517.1.S1	v-erb-b2 erythroblastic leukemia viral oncogene homolog 3 (avian)	2.43	0.0242
	Bt.21540.1.S1	fibroblast growth factor receptor 2	2.7	0.0119
	Bt.22053.1.S1	nuclear receptor binding protein 2	2.25	0.0271
	Bt.22649.1.A1	focal adhesion kinase	−3.82	0.0481
	Bt.4413.1.S1	diacylglycerol kinase, eta	3.05	0.0324
	Bt.729.1.S1	similar to TYRO3 protein tyrosine kinase	2.47	0.0119
	Bt.9194.1.S1	similar to microtubule associated serine/threonine kinase 2	2.42	0.0247
**Transcription or**	Bt.21228.1.A1	PAX interacting (with transcription-activation domain) protein 1	4.44	0.0323
**Translation**	Bt.1078.2.S1	Nuclear factor of activated T-cells, cytoplasmic	2.74	0.044
	Bt.20542.1.S1	Transcription factor jun-B	−2.17	0.0261
	Bt.20473.1.A1	similar to KIAA0833 protein	2.35	0.0175
	Bt.2418.1.S1	Similar to KIAA0934 protein	3.04	0.0324
	Bt.17848.1.S1	similar to transcriptional repressor BSR/RACK7/PRKCBP1	2.54	0.0126
	Bt.19585.1.S1	similar to TFIIH basal transcription factor complex p62 subunit	2.23	0.0276
	Bt.21110.1.S1	similar to neuroblastoma-amplified protein	3.94	0.0375
	Bt.4804.2.A1	Cyclin-dependent kinase inhibitor 1C (p57, Kip2)	2.33	0.0455
**Metabolism**	Bt.21917.1.S1	pyridoxal (pyridoxine, vitamin B6) phosphatase	2.76	0.0069
	Bt.3162.1.S1	**procollagen-proline, 2-oxoglutarate 4-dioxygenase**	2.21	0.0119
	Bt.23559.1.S1	similar to thiamin pyrophosphokinase 1	2.06	0.0119
	Bt.15925.1.S1	epoxide hydrolase 2, cytoplasmic	2.51	0.0349
	Bt.27130.1.S1	Saccharopine dehydrogenase	2.04	0.0212
	Bt.13710.1.S1	phosphatidylglycerophosphate synthase 1	3.17	0.049
	Bt.21376.1.S1	**STT3, subunit of the oligosaccharyltransferase complex, homolog**	2.24	0.0446
	Bt.20890.1.S1	amylase, alpha 2B (pancreatic)	3.38	0.0141
	Bt.24210.1.S1	acyl-CoA synthetase long-chain family member 1	2.22	0.0319
	Bt.1237.1.S1	A kinase (PRKA) anchor protein (yotiao) 9	2.11	0.0286
	Bt.25525.1.A1	Similar to Ectonucleotide pyrophosphatase/phosphodiesterase 2	2.72	0.0186
	Bt.1330.1.S1	aldo-keto reductase family 1, member B1 (aldose reductase)	2.87	0.0172
	Bt.22011.1.S1	O-linked N-acetylglucosamine (GlcNAc) transferase	2.12	0.0416
	Bt.5002.1.S1	glycerol-3-phosphate dehydrogenase 1	2.15	0.0447
	Bt.7951.1.S1	sphingomyelin phosphodiesterase 1, acid lysosomal	2.32	0.016
	Bt.9126.1.S1	similar to sterol-C5-desaturase-like	2.67	0.0441
	Bt.5517.1.S1	2′,3′-cyclic nucleotide 3′ phosphodiesterase	3.12	0.0387
	Bt.24519.1.S1	similar to holocarboxylase synthetase	2.25	0.0266
	Bt.25539.1.A1	similar to Heparan sulfate glucosamine 3-O-sulfotransferase 5	2.26	0.0209
	Bt.3284.2.A1	**Asparagine-linked glycosylation 3 homolog**	−2.02	0.0069
**DNA or RNA**	Bt.22982.1.A1	**reticulon 1**	2.56	0.0335
**binding**	Bt.10510.1.S1	H2A histone family, member X	2.49	0.0167
	Bt.26546.1.S1	MUS81 endonuclease homolog	3.76	0.0288
	Bt.22310.1.S1	ariadne homolog 2	2.28	0.0141
	Bt.22356.1.S1	AT rich interactive domain 1A	2.60	0.0482
	Bt.20959.1.S1	polymerase (DNA directed), alpha 2 (70 kD subunit)	−3.65	0.0373
	Bt.2594.1.S1	splicing factor, arginine/serine-rich 2	2.13	0.049
	Bt.15534.1.S1	tubulin, alpha 1	2.26	0.0355
	Bt.11182.2.S1	GC-rich sequence DNA-binding factor homolog	2.16	0.0052
	Bt.27445.1.A1	similar to ELAV-like protein 3	2.25	0.0476
	Bt.8206.1.S1	splicing factor, arginine/serine-rich 7, 35 kDa	2.05	0.0264
	Bt.13659.1.S1	similar to pre-mRNA processing 8 protein	2.0	0.0475
	Bt.13529.1.S1	similar to splicing factor 3b, subunit 1	2.1	0.0474
	Bt.15754.1.S1	similar to nucleolysin TIAR	2.16	0.0141
	Bt.18270.2.S1	similar to GW182 autoantigen	2.54	0.0209
	Bt.19937.1.S1	similar to carboxypeptidase D	2.18	0.0203
	Bt.20304.2.S1	similar to proliferation potential-related protein	2.53	0.0447
	Bt.21440.1.S1	similar to DEAD box polypeptide 17 isoform p82	2.1	0.0261
	Bt.28464.2.S1	splicing factor, arginine/serine-rich 4	2.92	0.0242
**Protein binding**	Bt.10723.1.S1	similar to RING finger protein 13	2.92	0.0129
	Bt.11149.1.S1	vimentin	2.38	0.0141
	Bt.20175.1.S1	HLA-B associated transcript 5	5.0	0.0175
	Bt.26104.1.A1	WAS protein family, member 1	2.17	0.0272
	Bt.13983.1.A1	metadherin	2.01	0.0399
	Bt.22603.1.S1	**leucine rich repeat transmembrane neuronal 1**	−2.56	0.036
	Bt.12039.1.S1	protein arginine methyltransferase 2	2.70	0.04
	Bt.18229.1.A1	similar to partner and localizer of BRCA2	4.22	0.0469
	Bt.12825.1.S1	similar to Actin, aortic smooth muscle (Alpha-actin-2)	4.54	0.049
	Bt.1690.1.S1	similar to goliath protein	2.05	0.0318
	Bt.1766.1.S1	actin related protein 2/3 complex subunit 1A	2.16	0.0467
	Bt.29710.1.A1	tight junction protein 3	−2.71	0.04
**Membrane**	Bt.10179.1.S1	liprin beta1	3.77	0.0375
**protein**	Bt.1076.1.S1	arylsulfatase A	2.20	0.0203
	Bt.5447.1.S1	natriuretic peptide receptor B/guanylate cyclase B	2.07	0.0105
	Bt.13265.1.A1	**similar to plasmolipin**	2.77	0.0165
	Bt.23589.2.S1	signal sequence receptor, alpha	2.19	0.0418
	Bt.22858.1.S1	uroplakin 3B	−2.3	0.0335
	Bt.5636.1.S1	**similar to Exocyst complex component 1**	2.07	0.0307
	Bt.13940.1.S1	similar to CDC91 cell division cycle 91-like 1	2.39	0.0206
	Bt.14205.1.S1	LMBR1 domain containing 1	2.57	0.0119
	Bt.3625.1.S1	transmembrane protein 85	2.16	0.0414
	Bt.15878.1.S1	similar to LanC-like protein 1 (40 kDa erythrocyte membrane protein)	3.24	0.0097
	Bt.20013.1.S1	similar to ELOVL family member 7, elongation of long chain fatty acids	2.5	0.0138
	Bt.20219.1.S1	similar to phosphatidyl inositol glycan class T	2.37	0.0385
	Bt.6405.1.S1	myelin basic protein	4.85	0.0303
	Bt.22251.1.A1	similar to chemokine-like factor super family 4	3.51	0.0385
	Bt.23354.1.S1	similar to epoxide hydrolase 1	2.63	0.0139
	Bt.5333.1.S1	cysteine-rich with EGF-like domains 1	2.31	0.0399
	Bt.2606.1.S1	lysosomal-associated membrane protein 2	3.17	0.0151
	Bt.3904.1.S1	thioredoxin-related transmembrane protein 2	2.99	0.0399
	Bt.488.1.S2	phospholipase A2 receptor 1, 180 kDa	2.26	0.0309
	Bt.24941.1.S1	abhydrolase domain containing 3	2.49	0.0242
	Bt.7172.1.S1	myelin oligodendrocyte glycoprotein	3.45	0.0052
	Bt.7677.1.S1	transmembrane protein 59-like	3.7	0.0475
	Bt.8787.1.S1	adiponectin receptor-1	2.6	0.0067
	Bt.3410.1.S1	synaptogyrin 2	2.48	0.0476
**Endosome**	Bt.11002.1.S1	OCIA domain containing 1	−4.92	0.0299
	Bt.11329.1.S1	vacuolar protein sorting 11 homolog	2.41	0.0385
	Bt.22508.1.S1	PRA1 domain family, member 2	3.09	0.0483
**Others**	Bt.12906.1.S1	similar to Gelsolin precursor (Actin-depolymerizing factor)	2.59	0.0209
	Bt.13321.1.S1	centrosome and spindle pole associated protein 1	2.26	0.0304
	Bt.14136.1.A1	similar to Endonuclease domain containing 1	3.52	0.0386
	Bt.21008.1.S1	similar to FGFR-like protein	2.76	0.0416
	Bt.22605.1.A1	zinc finger, CW type with PWWP domain 1	2.08	0.0178
	Bt.23348.2.S1	zyxin	3.83	0.0385
	Bt.26865.1.S1	Myosin head domain containing 1	5.75	0.0485
	Bt.1409.1.S1	tubulin polymerization-promoting protein family member 3	2.04	0.0236
	Bt.4386.1.S1	synapsin I	4.24	0.0482
	Bt.1088.1.S1	GTPase, IMAP family member 7	0.32	0.0419
	Bt.6989.1.S1	responsive to centrifugal force and shear	2.79	0.007
	Bt.28035.1.S1	**fat storage-inducing transmembrane protein 2**	3.64	0.0367

Gene functions are defined largely according to Affymetrix GO biological process term or GO molecular function term.

*: genes associated with the endoplasmic reticulum in bold.

Only 18 genes (8%) were down-regulated and 212 (92%) genes were up-regulated after the repeated or un-annotated probe sets were removed (Table 1). Each step of filtering was re-examined to determine the number of up and down-regulated genes. The 2 fold change filter yielded 2138 probe sets: 792 (37%) of them were down-regulated in BSE field cases and 1346 (63%) probe sets were up-regulated. When the 2 fold change and 1-way ANOVA filters were combined, 409 probe sets were selected: 366 (89%) of them up-regulated and 43 (11%) probe sets down-regulated. Therefore, the up-regulated genes were increased in percentage after the ANOVA filter.

The largest functional group amongst the 230 identified genes was the genes involved in transport (39 genes), followed by the membrane protein group (25 genes), the metabolism group (20 genes) and the DNA and RNA binding group (19 genes; Table 1). The maximal increase was 5.75 fold for Myosin head domain containing 1 and the maximal decrease was 4.92 fold for OCIA domain containing 1.

Many genes in Table 1 were linked to prion diseases in previous studies, such as cathepsin D [12], cathepsin B [13], Inositol 1,4,5-triphosphate receptor [13], apolipoprotein D [13], vimentin [12], [13], heat shock protein 70 [14], transferrin [15], reticulum 1 [16], reticulum 3 [17], a gene similar to solute carrier family 25 [13], CD9 [13], [18], vacuolar protein sorting 11 homolog [11] and DnaJ [11].

The microarray data were validated and confirmed by quantitative PCR using 5 genes: CD47, DnaJ, Hsp70 (up-regulated) and KCNB2, TNFRSF5 (down-regulated) (Figure S1).

**Figure 1:**
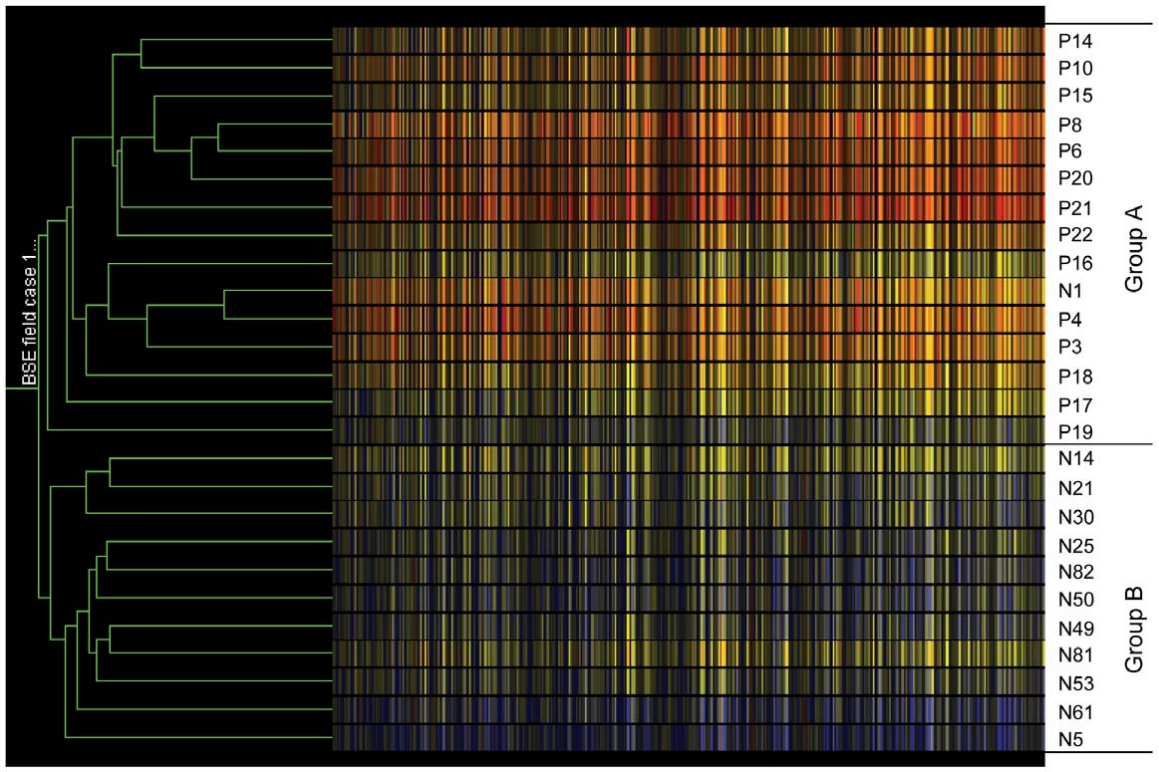
Condition tree of clustering analysis to test tissue sample consistence. The analysis was performed by GeneSpring using 409 differential regulated probe sets on Bovine GeneChips. The similarity was measured using the Spearman correlation with value 1 for separation ratio and value 0.001 for minimum distance in merge similar branches. N: negative controls and P: clinical BSE samples. Each of coloured bars represents a gene and the colour represents the levels of expression. The relative levels of expression are displayed in different colours: Red: 5; orange: 2; yellow: 1; dark yellow: 0.7; dark blue: 0.4; blue: 0.1.

Clustering analysis using the 409 probe sets showed that the samples were divided into two groups, Group A contained only negative control samples, while Group B contained all the BSE infected samples plus one negative control sample (Figure 1). This analysis confirmed that the samples of BSE and controls were relatively homogeneous amongst themselves with regard to the genes defined as differentially regulated.

### ER stress is implicated in disease pathogenesis

There were 17 differentially regulated genes whose products are associated with the ER (in bold in Table 1). Upregulation of glucose-regulated protein 94 (Grp94/gp96; ER stress response chaperone) and glucose-regulated protein 170 (Grp170/Orp150; ER stress response chaperone) suggests ER stress [19] as both of them are also known as ER stress markers (Table 1) [20]. Disturbance in the ER leads to ER stress which can be caused by accumulation of unfolded proteins and by changes in calcium homeostasis within the ER [21]. In BSE, many other stress related genes whose products are located in the ER were also up-regulated, such as Inositol 1,4,5-triphosphate receptor (IP3-R; ER calcium-depletion stress) [22]. reticulon 1 (ER stress induced apoptosis) [23], reticulon 3 (ER stress response) [24], reticulon 4 (ER stress induced apoptosis) [25], CDC91 cell division cycle 91-like (Gab1; oxidative stress) [26], procollagen-proline, 2-oxoglutarate 4-dioxygenase (P4HA1; ER stress response) [27], LAG1 homolog, ceramide synthase 2 (CerS2; inhibition of the unfolded protein response and autophagy) [28] and signal sequence receptor, alpha (SSR1 calcium binding) [29] (Table 1). In this study, both cytosolic chaperones Hsp70 and DnaJ were also found up-regulated (Table 1) and this chaperone pair is also induced by ER stress [30]. Other ER stress related gene products in the cytosol were: N-myc downstream regulated gene 1 (Ndrg1; ER stress responsive) [31], aldo-keto reductase family 1, member B1 (Akr1b1; anti ER stress) [32], O-linked N-acetylglucosamine (GlcNAc) transferase (anti ER stress) [33], transketolase (anti ER stress) [34] and cyclin-dependent kinase 5 (Cdk5; Apoptosis in ER stress) [35] (Table 1). These changes suggest the involvement of ER stress during BSE pathogenesis.

**Figure 2:**
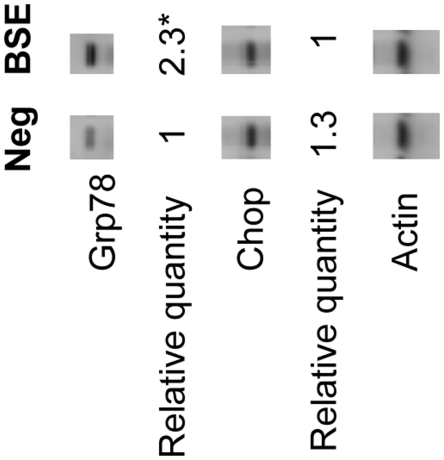
Western blotting of ER stress related proteins Grp78 and Chop. The relative quantity was the mean values of three controls (N5, N21 and N25) and three clinical BSE samples (P6, P10 and P14). β-Actin was used for normalization. *: p = 0.042 (student's t-test).

In response to ER stress, the unfolded protein response (UPR) is induced to restore cell function by reduction in newly translated proteins entering into the ER, by an increase in the capacity for protein folding [36]. If ER stress is prolonged, the UPR signaling pathways also initiate apoptosis [36]. In BSE, up-regulation of chaperones Grp94 and Grp170 suggests the induction of the UPR; while up-regulation of CerS2 indicates the inhibition of the UPR. To further explore the involvement of ER stress in the pathogenesis of BSE, Western blotting analysis on two more ER stress markers, Grp78 and Chop, was carried out. Grp78, is an ER chaperone and also known as an ER stress master regulator; while Chop is a transcription factor for induction of apoptosis, often up-regulated in response to ER stress [36]. In BSE, the Grp78 protein was up-regulated (Figure 2). Up-regulation of these ER chaperones: Grp78, 94 and 170 indicates the presence of ER stress and the activation of the UPR. The level of Chop was slightly decreased (Figure 2) and this is consistent with the evidence that apoptosis plays a very limited role in BSE [8].

### Using the gene expression profiles as a biomarker to represent BSE

**Figure 3:**
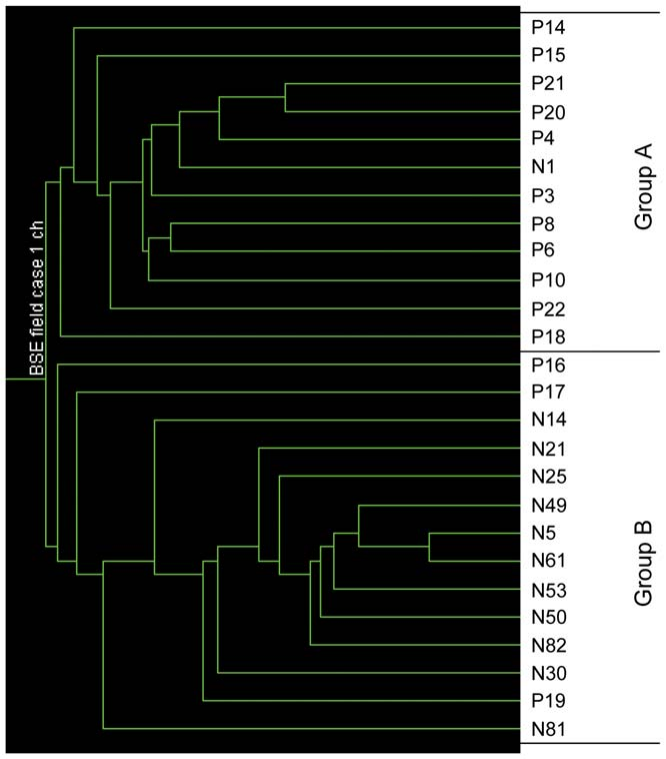
Clustering analysis of sample status in the BSE field case study. The analysis was performed by GeneSpring using 205 differential regulated probe sets generated from the time course study [11]. The similarity was measured using the spearman correlation. N: BSE negative controls; P: clinical BSE samples.

In our previous BSE time course study, 205 differentially regulated probe sets (corresponding to 114 genes) have been used to show that preclinical animals at 45 months post inoculation (mpi) cluster with cases positive for BSE and allowed the prediction that they are indeed preclinical and close to developing BSE [11]. The same 205 probes sets were used here in a clustering analysis to classify the disease status of the samples from the BSE field cases (Figure 3). These samples fell into two main groups: Group A contained 11 positives and one negative, Group B contained the remaining 11 negatives and three positives. This analysis was therefore able to classify the samples according to infection status with 78.5% (11/14) sensitivity and 92% (11/12) specificity.

**Figure 4:**
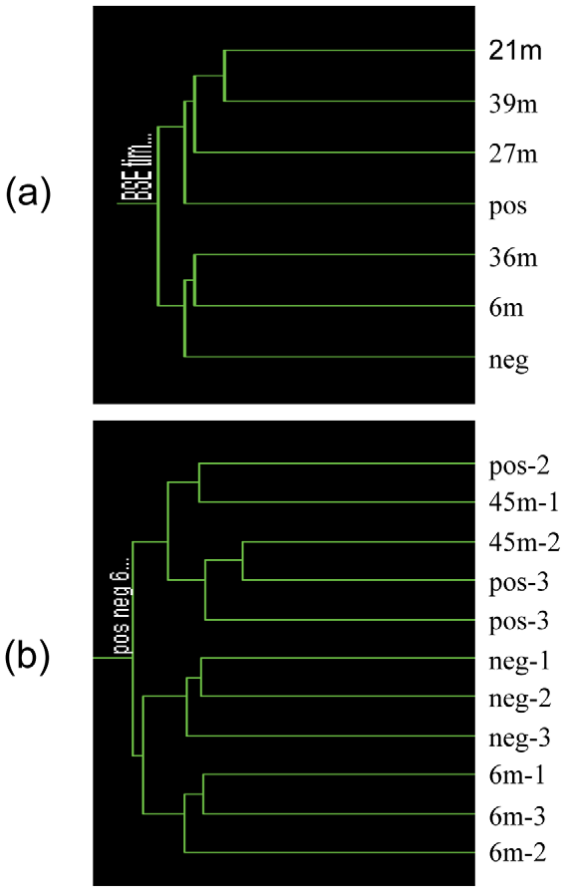
Clustering analysis of sample status in the BSE time course study. The analysis was performed by GeneSpring using 409 differential regulated probe sets generated from this study and the samples were from the BSE time course study [11]. The similarity was measured using the spearman correlation. Neg: BSE negative controls; Pos: clinical BSE samples and m: months post inoculation. (a): the samples were grouped to the time point; (b): the individual samples of negative controls, clinical BSE samples, 6 mpi and 45 mpi.

In a reverse analysis, the 409 probe sets identified in this study were used for clustering the samples from the time course study [11]. One group included the negatives, the samples from animals 6 mpi and 36 mpi and the other group contained the positives, and the samples from 21, 27 and 39 mpi animals (Figure 4a). The clustering was similar to the one derived with the 205 probe sets from the time course study [11]. When these 409 probe sets were used to predict the status of the preclinical animals at 45 mpi in the time course study the clustering analysis grouped the individual samples into two groups: one with all the negatives (n = 3) and 6 mpi samples (n = 3) and the other with all the positives (n = 3) and 45 mpi samples (n = 2) (Figure 4b).

**Figure 5:**
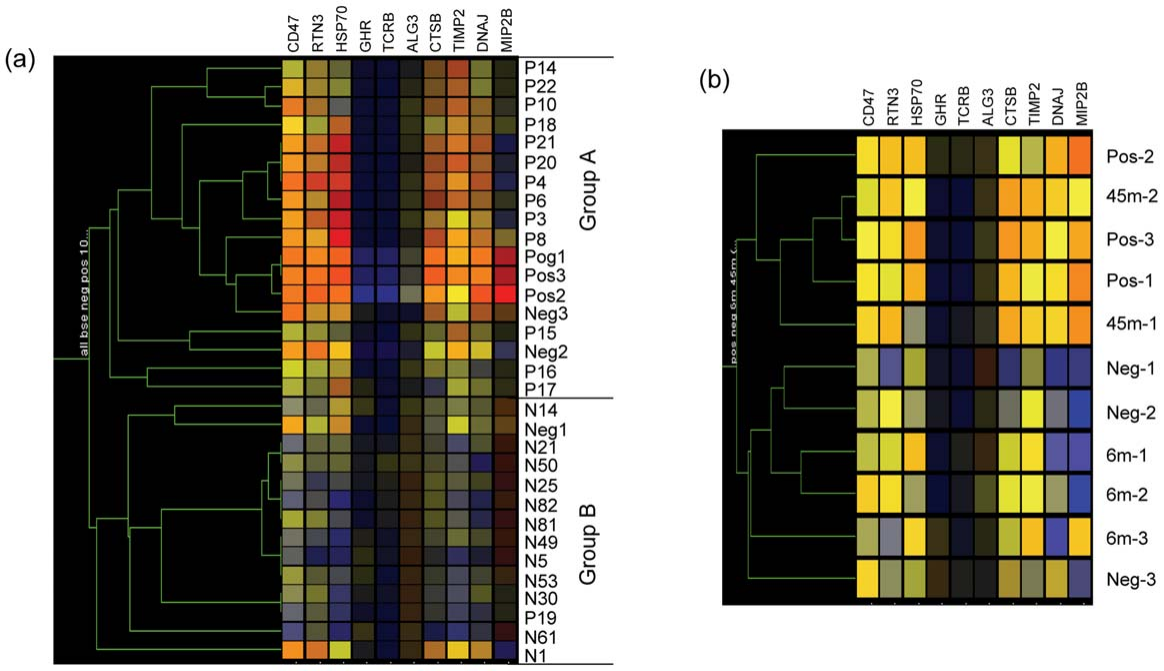
Clustering analysis of possible biomarkers for BSE. The analysis was performed by GeneSpring using 10 differential regulated genes generated from this study. The similarity was measured using the change correlation with value 1 for separation ratio and value 0.001 for minimum distance in merge similar branches. (a), All samples. N: BSE negative controls and P: clinical BSE samples in this study. Pos: clinical BSE and Neg: the negatives from the BSE time course study [11]. (b), samples from the BSE time course study. m: months post inoculation. Each of coloured bars represents a gene and colours represent the levels of expression. The relative levels of expression are displayed in different colours: Red: 5; orange: 2; yellow: 1; dark yellow: 0.7; dark blue: 0.4; blue: 0.1.

The analyses above indicate that either the genes from the time course study or the field case samples could be used to predict the infection status. However, it would not be practical to apply all 409 or 205 probe sets as biomarkers to represent BSE. A group of 10 genes were sought to represent BSE from these 230 genes listed in Table 1. Initially, the search was carried out using genes associated with prion diseases (10 genes), ER stress (10 genes), the largest fold changes (10 genes) or the smallest p values (10 genes) separately but the sensitivity and specificity of prediction were low. When these 40 genes were combined and10 genes were selected from them by comparing the expression levels of individual samples from both this study (clinical BSE, n = 14; control, n = 12) and the time course study (clinical BSE, n = 3; control, n = 3), only two groups were produced (Figure 5a). Group A contained all the clinical BSE samples from both studies and group B all the negatives with only three exceptions: P19, Neg2 and Neg3. The sensitivity of these biomarkers was 94% (16/17) and the specificity was 87% (13/15).

These 10 genes above were then used to classify the preclinical samples from the time course study with clustering analysis. The clustering analysis produced two groups: three negatives and three 6 mpi samples being one group and three positives and 45 mpi samples being the other with 100% (5/5) sensitivity and 100% (6/6) specificity (Figure 5b). Therefore, the results of these analyses suggest that these 10 genes might be used to represent the patterns of BSE gene expression at the terminal stages of BSE.

## Discussion

In this study, 230 genes were found to be differentially regulated between BSE field cases and controls (Table 1). These genes belong to many functional groups from apoptosis to transport. Seventeen genes were associated with the ER and 10 of them may be involved in stress related situations, especially up-regulation of ER chaperones Grp94 and Grp170 as they are ER stress markers. Since ER stress triggers the UPR [37], [38], [39], the level of protein expression of Grp78, another ER stress marker, was increased in BSE. Up-regulation of Grp78, Grp94 and Grp170 is induced by ER stress response transcription factors XBP1 and ATF6 as all three of them have an ER stress response element (ERSE) in their regulatory regions [36]. These analyses suggest the presence of ER stress and the activation of the UPR in the disease process of BSE. This is in agreement with increasing evidence of the involvement of ER stress in prion diseases [40], [41], [42]. In this study only changes in gene and protein expression of these chaperones were measured to indicate activation of the UPR. There are other methods to measure the induction of the UPR as many proteins are activated or inactivated through phosphorylation cascade in the UPR signalling pathways. For example, the release of Grp78 bound to PERK triggers autophosphorylation of PERK which in turn phosphorylates elf2α to attenuate protein translation [36].

To cope with accumulation of misfolded proteins, ER stress induces ER associated protein degradation I (ERAD I, ubiquitin/proteasome) [43] and ERAD II (autophagy/lysosome) [44], possibly through the UPR. ERAD I is closely linked to the ER quality control system [45] as unfolded or misfolded proteins are targeted for degradation after the failed attempt of folding by ER chaperones. In BSE, ubiquitin-activating enzyme E1 (UBE1) and three E3 ligases: WW domain containing E3 ubiquitin protein ligase 2 [46], ariadne homolog 2 [47] and ubiquitin carboxyl-terminal esterase L1 [48] were found to be up-regulated (Table 1). Recently, the E3 ligase HECTD2 has been identified as genetically associated with vCJD and kuru [49].

ERAD II is also known as autophagy. It is a pathway of self-degradation of cellular components in which autophagosomes sequester organelles or protein aggregates and fuse with lysosomes for degradation. When the production of misfolded proteins exceeds the capacity of ER chaperones and ERAD I, misfolded and aggregated proteins are targeted by the aggresome-autophagy pathway [50]. In BSE, up-regulation of several genes (Table 1) suggests that this pathway might be induced. In the lysosome, both cathepsin B and D (lysosomal hydroases) were up-regulated [44]. On the membrane of the lysosome, the increased levels of lysosomal-associated membrane protein 2 (LAMP2) suggest autophagy initiation [51]. In the cytosol, there were also several up-regulated genes related to ERAD II, such as ubiquitin carboxyl-terminal esterase L1 (aggresome initiation in proteasome inhibition) [48], sphingomyelin phosphodiesterase 1, acid lysosomal (SMPD1; autophagy promotion) [52] and vimentin (cytoskeleton) [14] (Table 1). This association between ERAD II and BSE has been shown in both mice and cattle [53], [54].

In this study, the analyses suggest that ER stress might be involved in BSE pathogenesis and that the UPR, ERAD I and II might all be activated in a concerted effort to rid the cell of harmful PrP^Sc^. The question, therefore, is how much these ER related pathogenic events contribute to fatal prion diseases in general. When the GPI anchor of the PrP protein is removed, the transgenic mice infected with scrapie, also a prion disease, can survive up to 400–600 days post infection (dpi) without clinical scrapie, while the wild type controls develop clinical signs within 140–160 dpi [55]. Some animals with this anchorless PrP have up to 40% more PrP^Sc^ than clinically sick controls. The results indicate that infectivity (PrP^Sc^ accumulation) and toxicity can be uncoupled. One model to explain it is intra neuronal generation of a toxic intermediate [32]. Here we offer another explanation of prion neurotoxicity using ER stress. The reason for PrP^Sc^ accumulation in the ER is because the ER quality control system senses the misfolded forms of PrP and ER chaperones retain them in the ER for folding or degradation by ERAD I. PrP^Sc^ is protease resistant so that the rate of removing the misfolded protein is slow; while more and more PrP^C^ converts to PrP^Sc^. Eventually, PrP^Sc^ accumulation causes ER stress and the subsequent activation of the UPR and ERAD I and II. Prolonged ER stress leads to cell death [45], [56]. Hence, ER stress related responses might be the major source of prion toxicity. What happens when misfolded PrP^Sc^ bypasses the ER quality control? There are lines of evidence that the anchorless prion protein is not detected by the ER quality control system [57], [58]. As the anchorless PrP^Sc^ can pass the ER efficiently, there is no toxicity to cause clinical scrapie. Since the cell is not under ER stress, the ERAD pathways are not activated. As a result, more PrP^Sc^ accumulates in the brain of transgenic mice with anchorless PrP than in the brain of the wild type controls.

**Figure 6:**
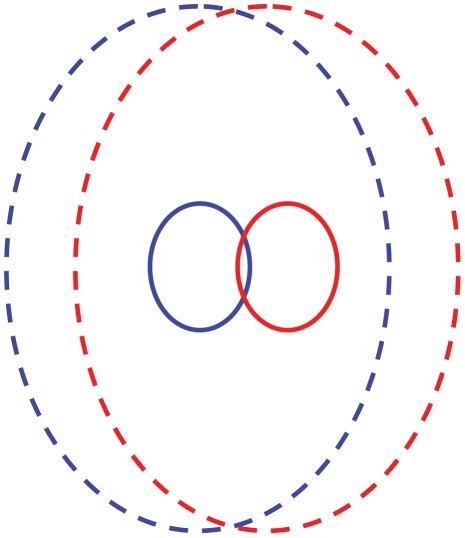
A model to explain the relationship between two BSE gene expression studies. The larger circles represent all differentially genes; while the inner circles represent differentially regulated genes listed in the studies with p value being less than 0.05. The blue circles: the time course study. The red circles: the field case study.

Both the current field case study and the previous time course study were carried out with brainstem tissues infected with BSE [11]. Although these two sets of samples differed in age, in infectious dose and in stages of disease development, many differentially regulated genes were expected to be shared between these two studies. Nonetheless, when the two gene lists were compared, there were only two genes overlapping. However, the profiles generated from one study could be used to predict the sample status of the other study as biomarkers, suggesting that there were some underlying links between these two gene lists (Figures 3 and 4). One possible explanation is that there are more differentially regulated genes than those identified by the analytical method. In order to define a gene list that is relevant with a condition or a disease within a study, the p value is often set at 0.05 or less. However, by doing so, much of the coverage is lost and many differentially regulated genes are not considered. In order to make the list more manageable, an additional 2 fold change filter was introduced to reduce the number of probe sets to 409. If the fold change filter had not been introduced and the p value had been set at 0.1, the number of probe sets would have been 1604. By definition, only 160 of them were selected randomly and the rest of 1446 probe sets should be truly differentially regulated. The remaining 1037 (1446-409) probe sets were not analyzed. Figure 6 provides a simple graphical model for this situation. The small inner circles (stringent settings) overlap only marginally. If all differentially regulated genes had been considered (large circles), there would have been many genes shared by these two studies and that is the most likely reason why the profiles from one study could be used to predict sample status from the other study. In recent years, there have been many publications on gene expression analyses of prion diseases. It is a surprise that relatively few differentially regulated genes are shared between these studies [11], [12], [13], [16], [59], [60]. However, the explanation above for the BSE studies may also apply to gene expression studies of prion diseases in general.

Considerable efforts have been made to find biomarkers for the prion diseases, especially in the early stage of the incubation period. To date, the detection of PrP^res^ is still the only reliable method. There are some reported potential biomarkers for the disease such as 14-3-3 protein [61], galectin-3 [62], SCRG1 [63], clusterin [64] and cystatin C [65]. However, none of them has been developed for routine diagnosis. One of the reasons may be the natural variation for the single marker within a population. Clustering analysis suggested that a prediction could be made by comparing the gene expression profiles of a sample with those of known BSE positive and negative samples. The analysis also showed a proof of principle that a prediction for a given sample could be made with high sensitivity (94%) and specificity (87%) using just 10 genes as biomarkers although the tissues used in this study were from the brainstem which may not be suitable for diagnose. These ten gene markers might represent the diseased state better than any single markers as they might allow some variations in expression. In Huntington's disease, gene expression profiling of blood reveals a subset of 12 up-regulated mRNAs which have been shown to be able to distinguish controls, presymptomatic Huntington's disease gene carriers and symptomatic Huntington's disease patients [66].

In conclusion, gene expression analysis suggests that BSE infection caused ER stress and the UPR, ERAD I and II might be induced in response to ER stress. Clustering analysis showed that the differentially regulated genes could be used to predict infection status. Ten genes were selected to represent gene expression state in BSE, which might eventually be used as biomarkers.

## Materials and Methods

### Tissue samples

Brainstem tissues from 100 confirmed cases of BSE in cattle were supplied by the TSE archive at the Veterinary Laboratories Agency, UK. The animals were females, between 4 and 10 years old that had been diagnosed clinically and killed on farm. The major breed was Holstein/Friesian and other breeds were: Limousin Cross, Guernsey, Hereford Cross and Brown Swiss. The negative controls (100 brainstem tissue samples) were from LGC Forensics (Queens Road, Teddington, Middlesex, TW11 0LY, UK) and were comparable in breed, sex and age with the naturally infected BSE samples. Since all samples were from the Archives, approval from the Ethics Committee was not necessary.

### Microarrays analysis

The preparations of samples and reagents were carried out according to the Affymetrix GeneChip Expression Analysis manual and as described in the previous study [11]. The RNA samples were resolved by 1% agarose gels and selected according to the integrity of ribosomal RNA bands. Since the tissues used in this study were from cattle naturally infected with BSE (field cases), the quality of RNA was generally poor. From 100 cases each, the best quality RNA samples, 12 controls and 14 BSE infected, were selected for microarray analysis with Affymetrix GeneChip Bovine Genome Arrays. The raw data were first imported into the Affymetrix GeneChip operating software version 1.4. All array data were MIAME compliant and the raw data were deposited in ArrayExpress with the accession number: E-MTAB-302. After initial analysis, the pivot formatted data were further analysed with the GeneSpring version 7 software (Silicon Genetics). The data were normalized in three steps: 1. Data transformation set measurements less than 0.01 to 0.01; 2. Each measurement was divided by the 50.0th percentile of all measurements in that sample; 3. Each measurement for each gene in test samples was divided by the median of that gene's measurements in the corresponding control samples. The value for each gene was divided by the median of its measurements in all samples. If the median of the raw values was below 10 then each measurement for that gene was divided by 10. If the numerator was above 10, the measurement was discarded. These steps were the default settings for the GeneSpring package.

Two filters were used to find differently regulated genes: 2 fold change and the one way ANOVA statistical analysis with the parameters of 0.05 for p-value cutoff, multiple testing correction and Student-Newman-Keuls for the *post hoc* tests, without assume variances equal for the parametric test.

### Western blotting

Cell-free extracts (60 µg protein) were loaded on 12% 1-D SDS PAGE (Invitrogen) and resolved proteins from several mini-gels were transferred to the same PVDF membrane (Millipore) so that one set of samples was used to monitor protein loading using β-Actin. The blots were immuno-stained with mouse monoclonal anti-β-Actin IgG (Santa Cruz Biotech), rabbit polyclonal anti-Grp78 (US Biological) and rabbit polyclonal anti-Chop (BioLegend). The protein bands were visualized by using secondary antibodies, alkaline phosphatase conjugated IgGs (anti-mouse, Santa Cruz Biotech; anti-rabbit, Sigma) and the ECL developer kit (Amersham). The images were captured by Fluor-S MultiImager (Bio-Rad) and the protein bands were quantified by the Quantity One software (Bio-Rad).

### Quantitative PCR

The RNA samples were treated with the DNA free™ kit (Ambion) for 1 h at 37°C to remove any trace of DNA. The treated RNA was then used as a template for cDNA synthesis with the TaqMan reverse transcription kit (Applied Biosystems). The real time PCR was carried out by denaturing at 95°C for 15 s, annealing at 50°C for 2 min and extension at 60°C for 1 min for 40 cycles using an ABI Prism 7700 Sequencing Detector. The GAPDH gene was used as an internal control to normalize the expression levels of target mRNA. The primer sets were chosen by the Primer Express 1.5 for TaqMan software. The sequences of the primer sets were as following: for CD47, 5′-TCC ATT AAC GAT TCT AAA TAA AGG AAA CT, 5′-TGC TAT GGA AAA AAG CCC CC and the probe, FAM-5′-TGG TGT TGC TAT GCG TGA GAT CCT CTC C; for DNAJ, 5′-TCT GTG AAA ATA AAG CAG GAG TGA A, 5′- AGT GAG AAA CAG CCA AAA TAC TGA AC and the probe, FAM-5′- CCT TTG CAG ACT TCA GAC TGG TTG GAT TTC; for KCNB2, 5′- TGA TGA CTT CTT AGA GCT CCA GGG, 5′-CAA GCA GTT TGG GCT GGA GT and the probe, FAM-5′-AGG AGG CCG GAC AAG CAG GCA; for HS70, 5′- GGA CTT TGG TCT TGC CCT ATA TTT AC, 5′-CAC ACT CAC TAT AAC ATA CAG AAA TAA CA AAA A and the probe, FAM-5′-TGT GAT GTG TCA GTT TGT TCT ATG ATA AGG TTG TAA TCT C; for TNFRSF5, 5′-CGT GGA GAC GAT TGA TCC G, 5′- AGC ATA AGG TCT CTT GCA CCG and the probe, FAM-5′- AGG ATT TTC CCG GCC CCC ACC.

## Supporting Information

Figure S1 Validation of microarray data by RT-PCR. 1 and 2: CD47; 3 and 4: DNAJ; 5 and 6: KCNB; 7 and 8: HS70; 9 and 10: TNFRSF5. The values of gene expression are listed at the top for comparison. No fill: negative controls; Grey: clinical BSE.
(0.24 MB TIF)

Table S1 Unannotated probe sets in the BSE field case study
(0.18 MB DOC)
