# Supplementary figures and images for: Transcriptional Analysis Implicates Endoplasmic Reticulum Stress in Bovine Spongiform Encephalopathy
Source: PLoS One. 2010 Dec 3;5(12):e14207. doi: 10.1371/journal.pone.0014207 (PMC2997050; doi:10.1371/journal.pone.0014207)

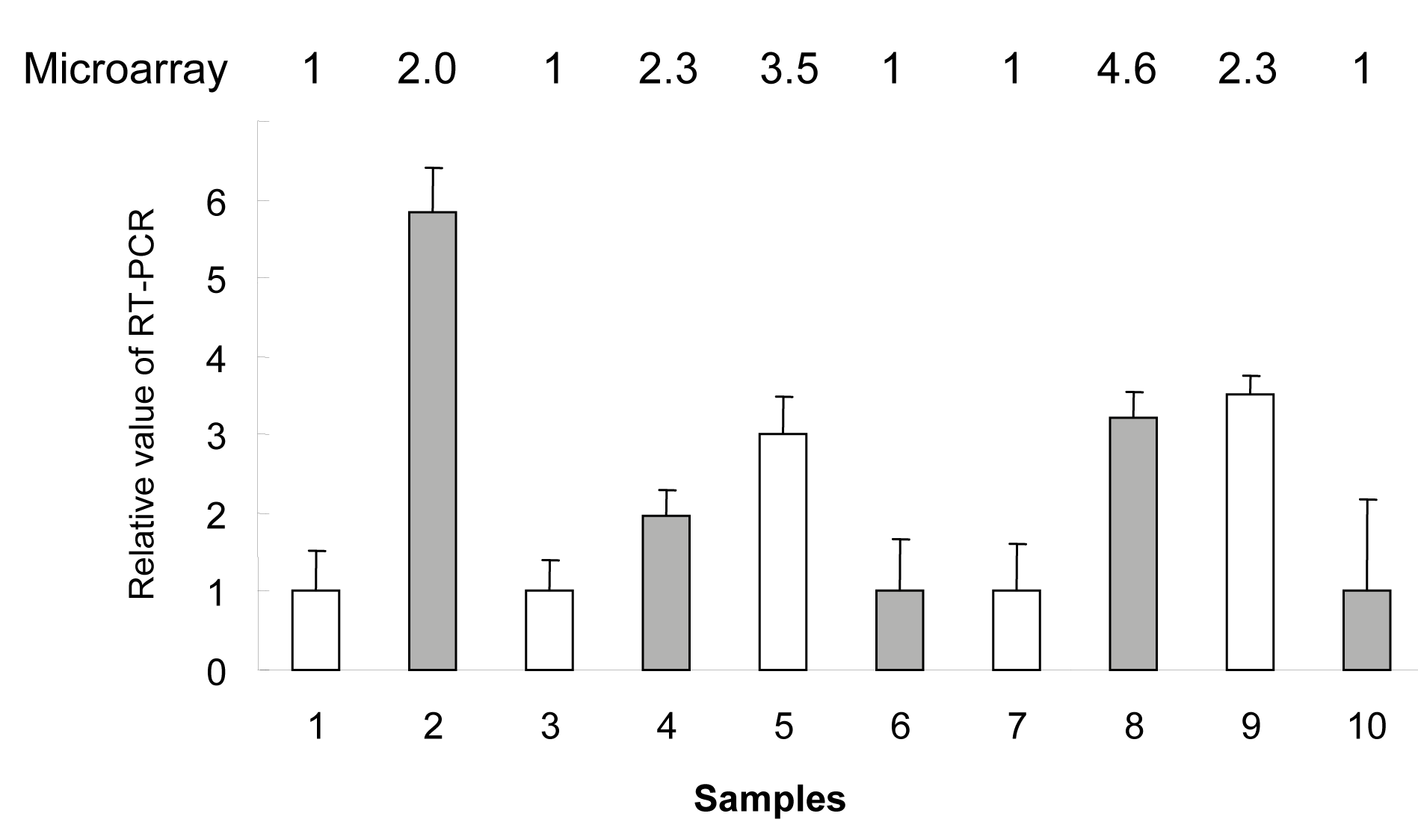

Supplement: Figure S1 — Validation of microarray data by RT-PCR. 1 and 2: CD47; 3 and 4: DNAJ; 5 and 6: KCNB; 7 and 8: HS70; 9 and 10: TNFRSF5. The values of gene expression are listed at the top for comparison. No fill: negative controls; Grey: clinical BSE. (0.24 MB TIF) [file pone.0014207.s001.tif]
