# Supplementary material for: Transcriptional Analysis Implicates Endoplasmic Reticulum Stress in Bovine Spongiform Encephalopathy
Source: PLoS One. 2010 Dec 3;5(12):e14207. doi: 10.1371/journal.pone.0014207 (PMC2997050; doi:10.1371/journal.pone.0014207)
Supplement: Table S1 — Unannotated probe sets in the BSE field case study (0.18 MB DOC) [file pone.0014207.s002.doc]

Table S1 Unannotated probe sets in the BSE field case study

| **Gene ID** | **fold change** | **p-value** |
| --- | --- | --- |
| Bt.10067.1.S1 | 2.41 | 0.0315 |
| Bt.1024.1.S1 | 3.62 | 0.0484 |
| Bt.11040.1.S1 | 2.74 | 0.0403 |
| Bt.11464.1.S1 | 2.45 | 0.0204 |
| Bt.11490.1.A1 | 3.30 | 0.0455 |
| Bt.11496.1.S1 | 4.93 | 0.0266 |
| Bt.12431.1.S1 | 2.20 | 0.0489 |
| Bt.12959.1.S1 | 3.12 | 0.0314 |
| Bt.13487.1.S1 | 2.38 | 0.0143 |
| Bt.13777.1.S1 | -4.25 | 0.035 |
| Bt.14370.1.A1 | -2.39 | 0.049 |
| Bt.15763.1.S1 | 2.12 | 0.0351 |
| Bt.16291.1.A1 | -2.43 | 0.0093 |
| Bt.17332.1.S1 | 3.14 | 0.0435 |
| Bt.17431.1.A1 | -3.53 | 0.041 |
| Bt.17514.1.S1 | -2.05 | 0.049 |
| Bt.1785.2.A1 | 2.20 | 0.0226 |
| Bt.18084.1.A1 | -3.65 | 0.0475 |
| Bt.18207.1.S1 | 2.45 | 0.02 |
| Bt.18207.2.S1 | 3.10 | 0.023 |
| Bt.1926.1.S1 | 2.26 | 0.0407 |
| Bt.1989.1.S1 | 2.42 | 0.0146 |
| Bt.20226.1.A1 | 2.10 | 0.0469 |
| Bt.20453.1.S1 | 2.22 | 0.0438 |
| Bt.21777.1.S1 | 2.14 | 0.0499 |
| Bt.23078.1.S1 | 2.60 | 0.0481 |
| Bt.23081.1.S1 | -3.85 | 0.0498 |
| Bt.23800.1.A1 | -4.79 | 0.041 |
| Bt.24127.1.A1 | -2.16 | 0.049 |
| Bt.24500.1.S1 | 2.54 | 0.049 |
| Bt.25612.1.A1 | -4.05 | 0.0467 |
| Bt.26847.1.S1 | -2.75 | 0.0423 |
| Bt.27111.1.A1 | 3.98 | 0.0203 |
| Bt.27550.1.A1 | -3.09 | 0.0237 |
| Bt.28602.1.S1 | 6.02 | 0.0329 |
| Bt.2962.1.S1 | 2.02 | 0.0275 |
| Bt.3261.1.S1 | 3.26 | 0.0186 |
| Bt.4136.1.S1 | 2.03 | 0.0141 |
| Bt.5580.1.S1 | 6.02 | 0.0368 |
| Bt.6591.1.S1 | 2.65 | 0.049 |
| Bt.6674.1.S1 | 2.69 | 0.0481 |
| Bt.7240.1.S1 | 2.65 | 0.0344 |
| Bt.7894.2.S1 | 3.33 | 0.041 |
| Bt.8182.1.S1 | 2.57 | 0.0444 |
| Bt.8433.1.S1 | 3.53 | 0.0209 |
| Bt.9131.1.S1 | -2.24 | 0.049 |
| Bt.9277.1.S1 | 3.69 | 0.049 |
| Bt.9576.3.S1 | -2.67 | 0.0474 |
| Bt.10047.1.S1 | 3.62 | 0.0475 |
| Bt.10092.1.S1 | 2.61 | 0.0481 |
| Bt.10109.1.S1 | -3.25 | 0.0272 |
| Bt.10122.1.S1 | 3.39 | 0.0288 |
| Bt.10135.1.A1 | 2.75 | 0.0138 |
| Bt.10222.1.S1 | 2.69 | 0.0119 |
| Bt.10236.1.S1 | 2.15 | 0.0482 |
| Bt.10631.1.A1 | 2.58 | 0.0431 |
| Bt.10734.1.A1 | 2.05 | 0.0209 |
| Bt.11059.1.S1 | 2.85 | 0.0084 |
| Bt.11356.1.A1 | -2.59 | 0.0455 |
| Bt.11998.1.S1 | 2.84 | 0.0209 |
| Bt.12080.1.S1 | 2.85 | 0.0154 |
| Bt.12179.1.S1 | 2.99 | 0.0182 |
| Bt.12348.1.A1 | 2.04 | 0.0272 |
| Bt.12586.1.A1 | 2.28 | 0.0407 |
| Bt.12609.1.A1 | 2.27 | 0.0272 |
| Bt.12872.1.S1 | 2.02 | 0.0164 |
| Bt.13402.1.S1 | 2.03 | 0.0261 |
| Bt.13833.1.S1 | 2.11 | 0.0382 |
| Bt.14043.2.S1 | 2.16 | 0.023 |
| Bt.14737.1.A1 | 2.06 | 0.0482 |
| Bt.1537.1.S1 | 2.18 | 0.0261 |
| Bt.15691.1.S1 | -3.23 | 0.0418 |
| Bt.15939.2.S1 | 3.77 | 0.02 |
| Bt.16265.1.S1 | 2.44 | 0.0141 |
| Bt.16717.1.A1 | -2.69 | 0.049 |
| Bt.1692.1.S1 | 2.44 | 0.0263 |
| Bt.1711.1.A1 | 3.50 | 0.0242 |
| Bt.17280.1.S1 | 3.02 | 0.0117 |
| Bt.17394.2.S1 | 2.65 | 0.0447 |
| Bt.17630.1.S1 | 2.10 | 0.0203 |
| Bt.1785.2.A1 | 2.57 | 0.0474 |
| Bt.17994.2.A1 | 4.92 | 0.0141 |
| Bt.18404.1.S1 | 4.29 | 0.0141 |
| Bt.18914.1.S1 | 2.60 | 0.0474 |
| Bt.19267.1.A1 | 3.22 | 0.0203 |
| Bt.19306.2.S1 | 2.58 | 0.0219 |
| Bt.19505.1.A1 | 5.56 | 0.0298 |
| Bt.19916.1.S1 | 2.88 | 0.0368 |
| Bt.20080.1.S1 | 3.47 | 0.0138 |
| Bt.20458.1.S1 | 2.44 | 0.0309 |
| Bt.21027.2.S1 | 2.48 | 0.0119 |
| Bt.21224.1.S1 | 2.90 | 0.0175 |
| Bt.21245.1.S1 | 2.92 | 0.0069 |
| Bt.21258.1.A1 | 2.01 | 0.049 |
| Bt.21307.1.S1 | 4.83 | 0.0423 |
| Bt.21376.1.S1 | 2.24 | 0.0446 |
| Bt.21383.2.S1 | 3.14 | 0.0055 |
| Bt.2144.2.S1 | 3.62 | 0.0247 |
| Bt.21873.1.S1 | 3.56 | 0.0112 |
| Bt.22190.1.S1 | 2.71 | 0.0288 |
| Bt.22286.1.S1 | 2.17 | 0.0273 |
| Bt.22319.1.S1 | 2.04 | 0.0424 |
| Bt.22470.1.S1 | 3.15 | 0.0069 |
| Bt.22552.1.S2 | 2.72 | 0.0385 |
| Bt.23216.1.S1 | 2.54 | 0.0249 |
| Bt.23331.1.S1 | 2.72 | 0.0057 |
| Bt.23548.2.S1 | 2.24 | 0.0328 |
| Bt.23616.1.S1 | 2.38 | 0.037 |
| Bt.23854.1.A1 | 3.38 | 0.0146 |
| Bt.23911.1.A1 | 4.59 | 0.016 |
| Bt.2416.1.S2 | 3.21 | 0.0137 |
| Bt.2425.1.A1 | 3.14 | 0.0378 |
| Bt.24370.1.A1 | 2.82 | 0.0397 |
| Bt.24514.1.A1 | 2.21 | 0.0356 |
| Bt.25051.1.A1 | 3.31 | 0.0134 |
| Bt.25202.1.A1 | 2.48 | 0.0282 |
| Bt.25207.1.A1 | 3.21 | 0.0324 |
| Bt.25735.1.A1 | -3.06 | 0.0212 |
| Bt.25915.1.A1 | 2.80 | 0.0249 |
| Bt.26014.1.A1 | 2.36 | 0.0475 |
| Bt.2616.1.S1 | 2.91 | 0.0382 |
| Bt.26223.1.S1 | 2.78 | 0.016 |
| Bt.2625.1.S1 | 3.76 | 0.0203 |
| Bt.26915.1.S1 | 2.31 | 0.0055 |
| Bt.27068.1.A1 | 3.13 | 0.0149 |
| Bt.27793.1.A1 | 2.35 | 0.0327 |
| Bt.27820.1.S1 | 2.44 | 0.0226 |
| Bt.2786.1.S1 | 2.23 | 0.0296 |
| Bt.28209.1.S1 | 2.31 | 0.049 |
| Bt.28321.1.A1 | 2.19 | 0.0141 |
| Bt.28617.1.S1 | 6.67 | 0.0226 |
| Bt.2889.1.S1 | 2.31 | 0.0141 |
| Bt.2911.1.A1 | 2.11 | 0.0442 |
| Bt.29372.1.A1 | -2.50 | 0.0328 |
| Bt.3123.1.A1 | 2.34 | 0.0306 |
| Bt.3526.1.S1 | 2.04 | 0.0237 |
| Bt.3803.1.A1 | 2.58 | 0.0212 |
| Bt.3825.1.S1 | 3.28 | 0.0164 |
| Bt.3880.1.S1 | 2.38 | 0.0133 |
| Bt.4010.1.S1 | 3.66 | 0.0055 |
| Bt.4228.1.S1 | 2.04 | 0.0475 |
| Bt.4392.2.A1 | 2.10 | 0.0164 |
| Bt.4474.2.S1 | 2.81 | 0.0288 |
| Bt.4715.1.S1 | 2.07 | 0.0339 |
| Bt.4827.1.S1 | 2.14 | 0.0070 |
| Bt.5144.1.S1 | 4.31 | 0.0288 |
| Bt.5604.1.A1 | 2.27 | 0.0335 |
| Bt.5633.1.S1 | 3.76 | 0.0399 |
| Bt.6553.1.A1 | 2.39 | 0.0324 |
| Bt.6733.1.S1 | 3.00 | 0.0226 |
| Bt.711.1.A1 | 2.42 | 0.0172 |
| Bt.9712.1.S1 | 2.28 | 0.0109 |
| Bt.9755.1.S1 | 2.06 | 0.0171 |

The probe sets are described as transcribed locus.
